# The Relationship between Serum Zinc Level and Heart Failure: A Meta-Analysis

**DOI:** 10.1155/2018/2739014

**Published:** 2018-02-25

**Authors:** Xuefang Yu, Lei Huang, Jinyan Zhao, Zhuoqun Wang, Wei Yao, Xianming Wu, Jingjing Huang, Bo Bian

**Affiliations:** ^1^Department of Cardiology, Tianjin Medical University General Hospital, Tianjin 300070, China; ^2^Department of Cardiology, Tianjin Union Medical Center, Tianjin 300121, China

## Abstract

Zinc is essential for the maintenance of normal cellular structure and functions. Zinc dyshomeostasis can lead to many diseases, such as cardiovascular disease. However, there are conflicting reports on the relationship between serum zinc levels and heart failure (HF). The purpose of the present study is to explore the relationship between serum zinc levels and HF by using a meta-analysis approach. PubMed, Web of Science, and OVID databases were searched for reports on the association between serum zinc levels and HF until June 2016. 12 reports with 1453 subjects from 27 case-control studies were chosen for the meta-analysis. Overall, the pooled analysis indicated that patients with HF had lower zinc levels than the control subjects. Further subgroup analysis stratified by different geographic locations also showed that HF patients had lower zinc levels than the control subjects. In addition, subgroup analysis stratified by HF subgroups found that patients with idiopathic dilated cardiomyopathy (IDCM) had lower zinc levels than the control subjects, except for patients with ischemic cardiomyopathy (ICM). In conclusion, the results of the meta-analysis indicate that there is a significant association between low serum zinc levels and HF.

## 1. Introduction

Zinc is an important trace element in the body. It plays a critical role in maintaining cellular structure and functions and is also involved in gene expression and cell growth and differentiation as a catalytic and structural cofactor [1]. Zinc deficiency accompanies many health conditions, such as renal disease, gastrointestinal disorders, alcoholism, sickle cell anemia, some cancer types, AIDS, and aging [2, 3]. The extracellular and intracellular levels of zinc are also related to cardiovascular health [4, 5]. According to Little et al., plasma zinc levels decrease with age and have a strong association with increasing cardiovascular disease (CVD); thus, there is an association between zinc deficiency and increased CVD [4]. Moreover, recent advances in cardiac biology and pathophysiology have highlighted the critical contribution of perturbations in zinc homeostasis to myocardial ischemia/reperfusion injury and the role of zinc signaling in cardioprotection against ischemia/reperfusion injury [2, 6].

Heart failure (HF) is one of the most frequent causes of death worldwide. Recently, there has been emerging evidence suggesting that micronutrient dyshomeostasis is associated with HF [7]. Many studies have attempted to explore the relationship between changes in serum zinc level in HF patients; however, conflicting results have been obtained. Some studies found significantly lower serum zinc levels in HF patients than in the control groups [8–16], whereas other works reported that the serum zinc levels were not significantly different between the HF patients and the control subjects [17–19]. Therefore, a comprehensive and critical meta-analysis of previous studies is carried out in the present work to draw a clearer, evidence-based conclusion on the association between serum zinc levels and HF.

## 2. Materials and Methods

### 2.1. Search Strategy

A systematic literature search of PubMed, Web of Science, and OVID databases was done until June 2016 with the use of medical subject headings (MeSH) or free text words. The search keywords were “zinc” or “Zn” and “heart failure.” The references cited in the studies and in review articles were also reviewed to identify additional works that were not captured by the database search. Only published studies with full-text reports were included.

### 2.2. Inclusion and Exclusion Criteria

Three authors (Xuefang Yu, Lei Huang, and Jinyan Zhao) carried out the search independently. Titles and abstracts were screened for subject relevance, and studies that could not be definitely excluded based on the abstract information were selected for full-text screening. Two authors (Xuefang Yu and Lei Huang) independently selected eligible studies for possible inclusion in the meta-analysis. Any disagreement regarding study inclusion was resolved by discussion with Jinyan Zhao toward reaching a consensus.

The appropriateness of the studies was assessed. The criteria for inclusion in the analysis were (1) human study, (2) case-control or cohort study or randomized clinical trial, (3) focus on the association between serum zinc levels and HF, and (4) providing sufficient data on zinc levels in both HF patients and control subjects. The exclusion criteria were (1) obviously irrelevant study, (2) animal study, (3) review or case report, and (4) not providing data on zinc levels in either HF patients or control subjects.

### 2.3. Data Extraction and Quality Assessment

All data were extracted independently by two reviewers (Bo Bian and Wei Yao) according to the inclusion and exclusion criteria. Specifically, the following data were extracted: authors' names, year of publication, country, number of subjects, and data on serum zinc levels. Any inconsistencies or discrepancies in the extracted information were resolved by discussion between the two reviewers, with a third author (Zhuoqun Wang) providing input.

The quality of all the included studies was evaluated by using the Newcastle-Ottawa Scale (NOS). This assessment tool focused on three aspects: participant selection, comparability, and exposure. Studies that satisfied all the items on the scale were given nine stars. Two authors (Xianming Wu and Jingjing Huang) assessed the quality of studies independently.

### 2.4. Statistical Analysis

The statistical analysis was carried out by using Stata 12, and statistical significance was set at *p* < 0.05. The standardized mean difference (SMD) and 95% confidence intervals (CI) were calculated. A random-effects or fixed-effects model was used to calculate the pooled SMD in the presence or absence of heterogeneity, respectively. Statistical heterogeneity was measured by applying chi-square and* I*-square tests. If the* I*^2^ value was less than 50% or the *p* value was greater than 0.10, significant heterogeneity was not considered, and a fixed-effects model was applied; otherwise, a random-effects model was used.

Subgroup analysis was done to determine associations between serum zinc levels and other relevant study characteristics, which may be possible sources of heterogeneity. Sensitivity analysis was carried out with one study removed at a time to assess whether the results could be affected markedly by a single study. Publication bias was measured by using Begg's test and visualization of funnel plots.

## 3. Results

**Figure 1:**
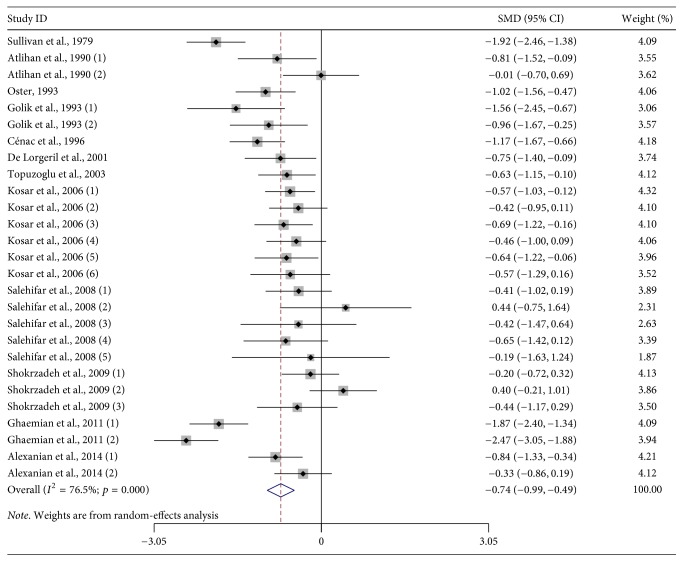
Forest plot of studies on zinc levels in HF patients versus control subjects. The combined SMD and 95% CIs were calculated by using a random-effects model.

Figure 1 shows the detailed steps of the literature selection. A total of 549 primary reports were identified by using the aforementioned search terms. After a series of assessments, 12 eligible articles with 1453 subjects from 27 case-control studies were chosen for the meta-analysis.  Table 1 presents the detailed characteristics of the included studies.

### 3.1. Serum Zinc Levels and HF

There were 12 studies that assessed the association between serum zinc levels and HF. First, the heterogeneity among the included studies was assessed. The results showed a high statistical heterogeneity (*I*^2^ = 76.5%; *p* < 0.001) (Table 2); thus, a random-effects model was used. The results of the random-effects meta-analysis indicated that HF patients had lower zinc levels than the control subjects [SMD: −0.740; 95% CI: −0.987, −0.493] (Figure 1).

### 3.2. Subgroup Analysis

The subgroup analysis showed that the geographic locations and etiologies of HF [idiopathic dilated cardiomyopathy (IDCM) and ischemic cardiomyopathy (ICM)] had some influence on the serum zinc levels in HF patients and control subjects.

**Figure 2:**
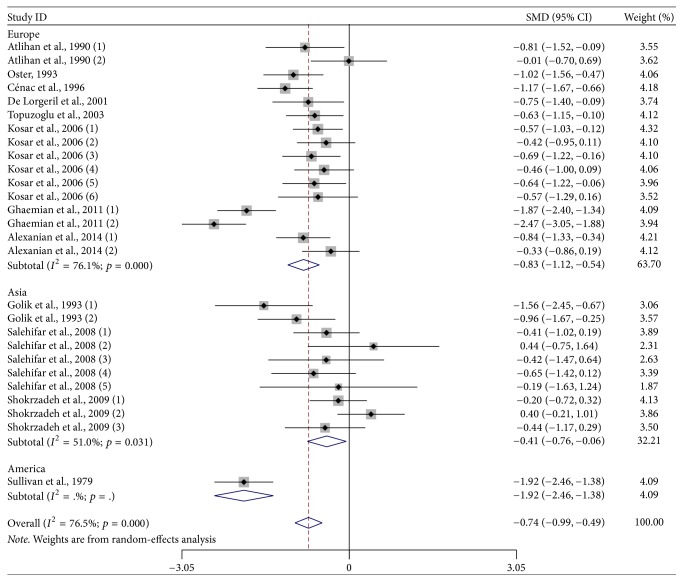
Subgroup analysis of studies on zinc levels in HF patients versus control subjects stratified by geographic location.

**Figure 3:**
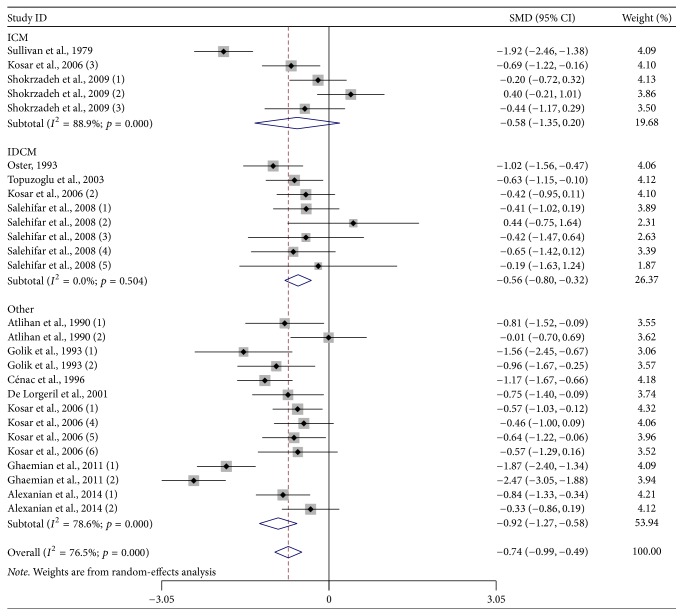
Subgroup analysis of studies on zinc levels in HF patients versus control subjects stratified by HF subgroups.

Further subgroup analysis stratified by different geographic locations found that HF patients had lower zinc levels than the control subjects [Europe: SMD: −0.832 and 95% CI: −1.119, −0.545; Asia: SMD: −0.408 and 95% CI: −0.761, −0.055; America: SMD: −1.920 and 95% CI: −2.456, −1.384] (Figure 2). In addition, the subgroup analysis stratified by HF subgroups found that patients with IDCM [SMD: −0.562; 95% CI: −0.804, −0.320] and other HF patients [SMD: −0.924; 95% CI: −1.267, −0.581] had lower zinc levels than the control subjects, except for ICM patients [SMD: −0.577; 95% CI: −1.353, 0.199] (Figure 3).  Table 3 provides a summary of subgroup analysis results.

### 3.3. Sensitivity Analysis and Publication Bias

**Figure 4:**
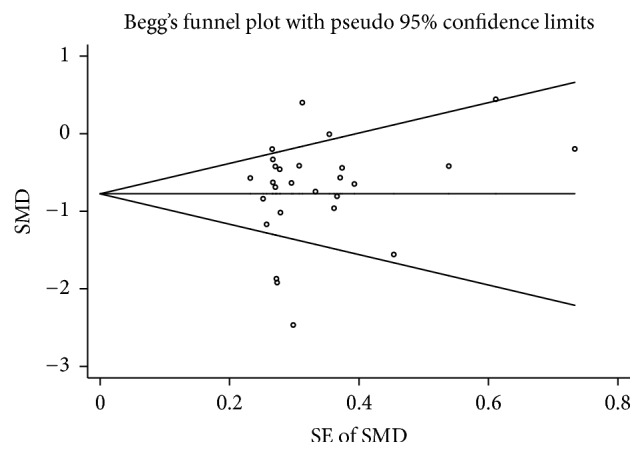
Funnel plot of studies on zinc levels in HF patients versus control subjects.

The sensitivity analysis showed that no individual study had an extreme influence on the pooled effect (Table 4). The publication bias was measured by using Begg's test, which showed no evidence of significant publication bias (*p* = 0.868), and by visualizing the funnel plot, which was symmetrical (Figure 4).

## 4. Discussion

Our meta-analysis included 12 reports with 1453 subjects from 27 case-control studies. The results showed that the serum zinc levels in HF patients were significantly lower than those in control subjects. This supported the supposition that there is some difference in zinc levels between HF patients and controls. In the subgroup analysis, the lower serum zinc levels in HF patients compared with control subjects were observed in different geographic locations (Europe, Asia, and America) and in IDCM and other HF patients. However, no significant difference in serum zinc levels was found in ICM patients, which could be due to the limited number of studies included in the analysis.

The mechanisms underlying the association between serum zinc levels and HF are still not fully understood. One underlying explanation for this meta-analysis showing is that micronutrient dyshomeostasis (such as zinc, copper, and Zn/Cu ratio dyshomeostasis) is associated with HF. The zinc and copper levels in the body affect each other, and decreased zinc levels are associated with deterioration of copper homeostasis and function [20]. For instance, decreased zinc levels, increased copper levels, and decreased Zn/Cu ratios have been observed in many diseases, such as rheumatoid arthritis, ICM, and thyroid carcinoma [19, 21, 22]. Moreover, a decreased Zn/Cu ratio and the subsequent systemic oxidative stress explained the more extensive atherosclerosis in some aged patients [23]. Indeed, previous studies have suggested that HF incidence and prevalence rates increased with advancing age [24]. Plasma zinc levels were found to decrease with age [3] and plasma cu to zinc ratio (CZr) was higher in hospitalized elderly subjects than in their healthy counterparts [25]. According to Malavolta et al., increments of CZr reflect increased inflammatory status or decreased nutritional status with subsequent appearance of some degenerative age-related diseases [26]. Thus, we speculated that lower levels of zinc in HF patients may be associated with advancing age or increasing of CZr due to age.

Zinc is an important component of superoxide dismutase (SOD). Manganese SOD and copper-zinc SOD, as metal-binding proteins and enzymes, have highly efficient antioxidant mechanisms and a preventive effect on the occurrence of free radical induced injury. Zinc deficiency has been found to be associated with lower SOD activity [27] and greater susceptibility to oxidative injury [28]. In HF, irrespective of the etiology, oxidative stress is important and correlates with the severity of symptoms and signs [29, 30]. Furthermore, some studies have reported a reduction of antioxidant defenses in HF patients [31–33]. Thus, in HF patients, these defenses (such as copper-zinc SOD) could be overwhelmed, creating an antioxidant deficit, particularly in cases when the activity of these oxidoreductases is dependent on the zinc concentrations.

According to Tousoulis et al., proinflammatory cytokines, such as interleukin-1 (IL-1), IL-6, and tumor necrosis factor-alpha (TNF-*α*), are elevated in states of HF and are related to long-term prognosis [34]. IL-1, IL-6, and TNF-*α* have been found to increase metallothioneins (MTs), which bind zinc from plasma and tissues, resulting in reduced zinc bioavailability [35]. Excessive catecholamine- and PTH-induced intracellular and intramitochondrial Ca^2+^ accumulation in HF has been found to be coupled with increased intracellular Zn^2+^ due to increased Zn^2+^ entry and nitric oxide-induced release of inactive Zn^2+^ bound to MT-1 [16, 36]. Thus, we speculated that elevated inflammatory markers in HF further induce MT-1 and consequently lower zinc levels.

In addition, HF may be associated with zinc deficiency through other mechanisms: reduced dietary intake (due to anorexia, nausea, and premature satiety with eating), reduced absorption due to gastrointestinal edema and impaired motility, increased intestinal zinc losses (protein-losing enteropathy), and excessive urinary excretion due to the use of diuretics [37]. Moreover, zinc deficiency frequently accompanies many health conditions, such as diabetes mellitus (DM), aging, and hypertension [2, 38]. The majority of HF patients are older, and many of them have various comorbidities, such as DM and hypertension, which may further impair zinc homeostasis. Cellular senescence was discovered as strong driver of atherosclerosis and heart failure [39, 40]. Recent observation performed on long-term culture of primary endothelial cells suggests that zinc deficiency may lead to accumulation of senescent cells and to vascular pathology as well as to heart failure [41]. Also, in HF patients, treatment with angiotensin-converting enzyme (ACE) inhibitors and angiotensin receptor blockers (ARBs) results in increased urinary zinc excretion and zinc deficiency [37].

To the best of our knowledge, this is the first meta-analysis to evaluate the association between zinc levels and HF. The sensitivity analysis showed that excluding any study from the pooled analysis did not vary the results substantially. Publication bias was also absent, as determined by funnel plot visualization and Begg's test. However, the possible limitations of our study should be considered. First, some too-old studies were included in the meta-analysis, which might weaken the quality of the results. Second, heterogeneity could not be eliminated because of methodological diversity between studies; thus, the conclusion should be conservative. In addition, because of limited data, we did not analyze the zinc levels in other tissues; this might affect the comprehensive interpretation of the zinc levels in HF patients. Therefore, better designed studies are required to verify the results and further assess the role of zinc in the progress of HF.

## 5. Conclusions

In conclusion, the results of the meta-analysis indicate that there is a significant association between low serum zinc levels and HF. Meanwhile better designed studies are required to verify the results and further assess the role of zinc in the progress of HF.

## Figures and Tables

**Table 1:** Characteristics of subjects in included studies.

Studies	Country	Number of participants		Age (year)		Subgroups of HF	Score
		HF	Controls	HF	Controls		
Sullivan et al., 1979	USA	42	37	NA	NA	ICM	6
Atlihan et al., 1990 (1)	Turkey	29	11	2.3 ± 1.5	3.1 ± 2.8	NA	6
Atlihan et al., 1990 (2)	Turkey	29	11	2.3 ± 1.5	3.1 ± 2.8	NA	6
Oster, 1993	Germany	20	50	53 ± 8	50.5 ± 7.2	IDCM	8
Golik et al., 1993 (1)	Israel	9	20	63.8 ± 6	48.7 ± 8	NA	8
Golik et al., 1993 (2)	Israel	15	20	64.9 ± 5	48.7 ± 8	NA	8
Cénac et al., 1996	France	35	36	NA	NA	NA	7
De Lorgeril et al., 2001	*France*	21	18	27–76	34–68	CHF	7
Topuzoglu et al., 2003	Turkey	54	20	18–75	21–73	IDCM	6
Kosar et al., 2006 (1)	Turkey	54	30	62 ± 10	56 ± 8	CHF	8
Kosar et al., 2006 (2)	Turkey	26	30	62 ± 10	56 ± 8	IDCM	8
Kosar et al., 2006 (3)	Turkey	28	30	62 ± 10	56 ± 8	ICM	8
Kosar et al., 2006 (4)	Turkey	24	30	62 ± 10	56 ± 8	NA	8
Kosar et al., 2006 (5)	Turkey	20	30	62 ± 10	56 ± 8	NA	8
Kosar et al., 2006 (6)	Turkey	10	30	62 ± 10	56 ± 8	NA	8
Salehifar et al., 2008 (1)	Iran	18	27	49.06 ± 8.88	42.30 ± 8.99	IDCM	8
Salehifar et al., 2008 (2)	Iran	3	27	49.06 ± 8.88	42.30 ± 8.99	IDCM	8
Salehifar et al., 2008 (3)	Iran	4	27	49.06 ± 8.88	42.30 ± 8.99	IDCM	8
Salehifar et al., 2008 (4)	Iran	9	27	49.06 ± 8.88	42.30 ± 8.99	IDCM	8
Salehifar et al., 2008 (5)	Iran	2	27	49.06 ± 8.88	42.30 ± 8.99	IDCM	8
Shokrzadeh et al., 2009 (1)	Iran	30	27	57.17 ± 8.88	42.3 ± 8.99	ICM	7
Shokrzadeh et al., 2009 (2)	Iran	17	27	57.17 ± 8.88	42.3 ± 8.99	ICM	7
Shokrzadeh et al., 2009 (3)	Iran	10	27	57.17 ± 8.88	42.3 ± 8.99	ICM	7
Ghaemian et al., 2011 (1)	Turkey	38	40	70.1 ± 9.2	64.9 ± 4.7	NA	8
Ghaemian et al., 2011 (2)	Turkey	40	40	66.7 ± 11.5	64.9 ± 4.7	NA	8
Alexanian et al., 2014 (1)	Greece	81	21	69.22 ± 11.09	57.0 ± 19.11	NA	8
Alexanian et al., 2014 (2)	Greece	44	21	67.50 ± 11.17	57.0 ± 19.11	NA	8

**Table 2:** Summary of studies included in the analysis of serum zinc levels for subjects with HF versus control subjects.

Author	HF		Controls		Weight (%)	SMD (95% CI)
	Number	Zn concentration (mean ± SD)	Number	Zn concentration (mean ± SD)		
Sullivan et al., 1979	42	0.74 ± 0.11 *μ*g/ml	37	0.97 ± 0.13 *μ*g/ml	4.09	−1.920 (−2.456, −1.384)
Atlihan et al., 1990 (1)	29	92.9 ± 18.9 *µ*g/100 ml	11	107.5 ± 15.7 *µ*g/100 ml	3.55	−0.806 (−1.523, −0.089)
Atlihan et al., 1990 (2)	29	107.4 ± 17.2 *µ*g/100 ml	11	107.5 ± 15.7 *µ*g/100 ml	3.62	−0.006 (−0.700, 0.688)
Oster, 1993	20	745 ± 195 *µ*g/l	50	931 ± 178 *µ*g/l	4.06	−1.017 (−1.563, −0.4710)
Golik et al., 1993 (1)	9	11.5 ± 1.53 *μ*mol/L	20	16 ± 3.3 *μ*mol/L	3.06	−1.557 (−2.446, −0.667)
Golik et al., 1993 (2)	15	13.2 ± 2.3 *μ*mol/L	20	16 ± 3.3 *μ*mol/L	3.57	−0.960 (−1.668, −0.251)
Cénac et al., 1996	35	0.9 ± 0.21 *μ*g/ml	36	1.17 ± 0.25 *μ*g/ml	4.18	−1.168 (−1.672, −0.664)
De Lorgeril et al., 2001	21	0.82 ± 0.12 mg/l	18	0.9 ± 0.09 mg/l	3.74	−0.746 (−1.398, −0.094)
Topuzoglu et al., 2003	54	81.42 ± 15.4 *µ*g/dl	20	92.51 ± 22.8 *µ*g/dl	4.12	−0.628 (−1.151, −0.105)
Kosar et al., 2006 (1)	54	555 ± 104 *µ*g/l	30	620 ± 130 *µ*g/l	4.32	−0.571 (−1.026, −0.116)
Kosar et al., 2006 (2)	26	568 ± 116 *µ*g/l	30	620 ± 130 *µ*g/l	4.1	−0.420 (−0.951, 0.111)
Kosar et al., 2006(3)	28	542 ± 92 *µ*g/l	30	620 ± 130 *µ*g/l	4.1	−0.689 (−1.219, −0.158)
Kosar et al., 2006 (4)	24	565 ± 107 *µ*g/l	30	620 ± 130 *µ*g/l	4.06	−0.457 (−1.001, 0.087)
Kosar et al., 2006 (5)	20	543 ± 106 *µ*g/l	30	620 ± 130 *µ*g/l	3.96	−0.636 (−1.216, −0.056)
Kosar et al., 2006 (6)	10	553 ± 66 *µ*g/l	30	620 ± 130 *µ*g/l	3.52	−0.568 (−1.295, 0.159)
Salehifar et al., 2008 (1)	18	0.97 ± 0.25 mg/l	27	1.12 ± 0.42 mg/l	3.89	−0.414 (−1.017, 0.189)
Salehifar et al., 2008 (2)	3	1.3 ± 0.09 mg/l	27	1.12 ± 0.42 mg/l	2.31	0.444 (−0.754, 1.642)
Salehifar et al., 2008 (3)	4	0.95 ± 0.26 mg/l	27	1.12 ± 0.42 mg/l	2.63	−0.418 (−1.474, 0.637)
Salehifar et al., 2008 (4)	9	0.87 ± 0.24 mg/l	27	1.12 ± 0.42 mg/l	3.39	−0.649 (−1.419, 0.121)
Salehifar et al., 2008 (5)	2	1.04 ± 0.06 mg/l	27	1.12 ± 0.42 mg/l	1.87	−0.194 (−1.631, 1.243)
Shokrzadeh et al., 2009 (1)	30	1.05 ± 0.28 mg/l	27	1.12 ± 0.42 mg/l	4.13	−0.198 (−0.719, 0.323)
Salehifar et al., 2009 (2)	17	1.27 ± 0.29 mg/l	27	1.12 ± 0.42 mg/l	3.86	0.399 (−0.214, 1.012)
Shokrzadeh et al., 2009 (3)	10	0.95 ± 0.27 mg/l	27	1.12 ± 0.42 mg/l	3.5	−0.439 (−1.172, 0.294)
Ghaemian et al., 2011 (1)	38	24.7 ± 27.6 *µ*g/dl	40	70.9 ± 21.6 *µ*g/dl	4.09	−1.870 (−2.405, −1.336)
Ghaemian et al., 2011 (2)	40	23.2 ± 16.8 *µ*g/dl	40	70.9 ± 21.6 *µ*g/dl	3.94	−2.465 (−3.050, −1.881)
Alexanian et al., 2014 (1)	81	74.27 ± 15.87 *µ*g/dl	21	87.9 ± 17.85 *µ*g/dl	4.21	−0.837 (−1.331, −0.343)
Alexanian et al., 2014 (2)	44	81.51 ± 19.73 *µ*g/dl	21	87.9 ± 17.85 *µ*g/dl	4.12	−0.334 (−0.857, 0.189)

**Table 3:** Subgroup analyses of zinc level and heart failure (HF).

Subgroup	Numberof studies	SMD (95% CI)	Test of SMD = 0		Heterogeneity	
			*Z*	*p* for *Z*	*I* ^2^	*p* for *I*^2^
Geographical location						
Europe	16	−0.832 (−1.119, −0.545)	<0.001	5.68	76.1%	<0.001
Asia	10	−0.408 (−0.761, −0.055)	2.27	0.023	51.0%	0.031
America	1	−1.920 (−2.456, −1.384)	<0.001	7.02	NA	NA
Subgroups of HF						
ICM patients	5	−0.577 (−1.353, 0.199)	1.46	0.145	88.9%	<0.001
IDCM patients	8	−0.562 (−0.804, −0.320)	4.55	<0.001	0.0%	0.504
Other HF patients	14	−0.924 (−1.267, −0.581)	5.28	<0.001	78.6%	<0.001

**Table 4:** The heterogeneity of the included studies through sensitivity analysis.

Excluded studies	SMD (95% CI)	*I* ^2^	*p* value
Sullivan et al., 1979	−0.692 (−0.929, −0.456)	72.90%	<0.001
Atlihan et al., 1990 (1)	−0.737 (−0.993, −0.482)	77.40%	<0.001
Atlihan et al., 1990 (2)	−0.768 (−1.018, −0.518)	76.30%	<0.001
Oster, 1993	−0.728 (−0.985, −0.470)	77.20%	<0.001
Golik et al., 1993 (1)	−0.715 (−0.965, −0.464)	76.70%	<0.001
Golik et al., 1993 (2)	−0.732 (−0.987, −0.476)	77.30%	<0.001
Cénac et al., 1996	−0.721 (−0.977, −0.465)	76.90%	<0.001
De Lorgeril et al., 2001	−0.739 (−0.996, −0.483)	77.40%	<0.001
Topuzoglu et al., 2003	−0.744 (−1.002, −0.486)	77.30%	<0.001
Kosar et al., 2006 (1)	−0.747 (−1.006, −0.487)	77.20%	<0.001
Kosar et al., 2006 (2)	−0.753 (−1.010, −0.497)	77.00%	<0.001
Kosar et al., 2006 (3)	−0.741 (−1.000, −0.483)	77.40%	<0.001
Kosar et al., 2006 (4)	−0.752 (−1.008, −0.495)	77.10%	<0.001
Kosar et al., 2006 (5)	−0.744 (−1.001, -0.486)	77.30%	<0.001
Kosar et al., 2006 (6)	−0.746 (−1.001, −0.491)	77.30%	<0.001
Salehifar et al., 2008 (1)	−0.753 (−1.008, −0.497)	77.10%	<0.001
Salehifar et al., 2008 (2)	−0.769 (−1.016, −0.521)	76.50%	<0.001
Salehifar et al., 2008 (3)	−0.749 (−1.001, −0.496)	77.30%	<0.001
Salehifar et al., 2008 (4)	−0.743 (−0.998, −0.488)	77.40%	<0.001
Salehifar et al., 2008 (5)	−0.751 (−1.001, −0.500)	77.20%	<0.001
Shokrzadeh et al., 2009 (1)	−0.764 (−1.017, −0.511)	76.30%	<0.001
Salehifar et al., 2009 (2)	−0.788 (−1.028, −0.549)	73.90%	<0.001
Salehifar et al., 2009 (3)	−0.751 (−1.005, −0.496)	77.20%	<0.001
Ghaemian et al., 2011 (1)	−0.694 (−0.932, −0.456)	73.30%	<0.001
Ghaemian et al., 2011 (2)	−0.676 (−0.891, −0.460)	67.60%	<0.001
Alexanian et al., 2014 (1)	−0.735 (−0.994, −0.476)	77.40%	<0.001
Alexanian et al., 2014 (2)	−0.757 (−1.013, −0.502)	76.80%	<0.001
